# A Framework (SOCRATex) for Hierarchical Annotation of Unstructured Electronic Health Records and Integration Into a Standardized Medical Database: Development and Usability Study

**DOI:** 10.2196/23983

**Published:** 2021-03-30

**Authors:** Jimyung Park, Seng Chan You, Eugene Jeong, Chunhua Weng, Dongsu Park, Jin Roh, Dong Yun Lee, Jae Youn Cheong, Jin Wook Choi, Mira Kang, Rae Woong Park

**Affiliations:** 1 Department of Biomedical Sciences Ajou University Graduate School of Medicine Suwon Republic of Korea; 2 Department of Preventive Medicine and Public Health Yonsei University College of Medicine Seoul Republic of Korea; 3 Department of Biomedical Informatics Vanderbilt University School of Medicine Nashville, TN United States; 4 Department of Biomedical Informatics Columbia University New York, NY United States; 5 Department of Biomedical Informatics Ajou University School of Medicine Suwon Republic of Korea; 6 Department of Pathology Ajou University Hospital Suwon Republic of Korea; 7 Department of Gastroenterology Ajou University School of Medicine Suwon Republic of Korea; 8 Department of Radiology Ajou University School of Medicine Suwon Republic of Korea; 9 Department of Digital Health Samsung Advanced Institute for Health Sciences & Technology Sungkyunkwan University Seoul Republic of Korea

**Keywords:** natural language processing, search engine, data curation, data management, common data model

## Abstract

**Background:**

Although electronic health records (EHRs) have been widely used in secondary assessments, clinical documents are relatively less utilized owing to the lack of standardized clinical text frameworks across different institutions.

**Objective:**

This study aimed to develop a framework for processing unstructured clinical documents of EHRs and integration with standardized structured data.

**Methods:**

We developed a framework known as Staged Optimization of Curation, Regularization, and Annotation of clinical text (SOCRATex). SOCRATex has the following four aspects: (1) extracting clinical notes for the target population and preprocessing the data, (2) defining the annotation schema with a hierarchical structure, (3) performing document-level hierarchical annotation using the annotation schema, and (4) indexing annotations for a search engine system. To test the usability of the proposed framework, proof-of-concept studies were performed on EHRs. We defined three distinctive patient groups and extracted their clinical documents (ie, pathology reports, radiology reports, and admission notes). The documents were annotated and integrated into the Observational Medical Outcomes Partnership (OMOP)-common data model (CDM) database. The annotations were used for creating Cox proportional hazard models with different settings of clinical analyses to measure (1) all-cause mortality, (2) thyroid cancer recurrence, and (3) 30-day hospital readmission.

**Results:**

Overall, 1055 clinical documents of 953 patients were extracted and annotated using the defined annotation schemas. The generated annotations were indexed into an unstructured textual data repository. Using the annotations of pathology reports, we identified that node metastasis and lymphovascular tumor invasion were associated with all-cause mortality among colon and rectum cancer patients (both *P*=.02). The other analyses involving measuring thyroid cancer recurrence using radiology reports and 30-day hospital readmission using admission notes in depressive disorder patients also showed results consistent with previous findings.

**Conclusions:**

We propose a framework for hierarchical annotation of textual data and integration into a standardized OMOP-CDM medical database. The proof-of-concept studies demonstrated that our framework can effectively process and integrate diverse clinical documents with standardized structured data for clinical research.

## Introduction

### Background

With the universal adoption of electronic health records (EHRs), the secondary use of EHRs becomes important for translational research and improvement of the quality of health care [1-3]. EHRs comprise structured (ie, diagnoses, medications, procedures, laboratory tests, and medical device use) and unstructured records, such as clinical notes with diverse formats. Structured data have been widely utilized owing to their processable and standardized codes. In an international open science initiative, Observational Health Data Sciences and Informatics (OHDSI), the structured data of more than 200 hospitals worldwide were mapped into a standardized vocabulary and data structure referred to as the Observational Medical Outcomes Partnership (OMOP)-common data model (CDM) [4]. OHDSI is an open collaborative research community, and researchers from each country have collaborated for discovering medical knowledge. OMOP-CDM version 6.0 consists of 15 clinical data tables, four health system data tables, two health economics data tables, three derived tables, and 10 vocabulary tables. All of the tables are represented with standardized medical terminologies. Using the OMOP-CDM, OHDSI has generated medical evidence through large-scale observational research [5], which can be achieved by the software and user interface to facilitate standardized phenotyping [6], statistical analysis [7], and machine-learning application [8].

Clinical notes with natural language are keeping invaluable information that is not in available structured data, such as clinician’s thoughts and medical profiles [9,10]. Although textual data can complement structured data and provide reliable clinical evidence, consistently processing textual data across multiple hospitals has been profoundly restricted. To process unstructured textual data, natural language processing (NLP) technology, an area of computer science for transforming human linguistics into a machine-readable form, is required [11-13]. Clinical documents in the OMOP-CDM have not been actively used for research in OHDSI because of difficulties in consistently processing the textual data and lack of standardized text mining pipelines. Therefore, a standardized clinical text framework for extracting, processing, and annotating unstructured clinical documents is essential to maximize the usefulness of the large body of clinical data in the OMOP-CDM format around the world.

One of the primary streams of clinical NLP is named entity recognition (NER), which extracts information of interest based on annotation schemas [14]. However, most NER studies have used a relatively narrow schema that permits restricted relationships and categories of medical concepts. The restricted medical concepts indicate that only limited information can be extracted from the narratives [15,16]. Conversely, hierarchical annotation leverages a multilevel data structure to extract a wide range of information. Users can richly annotate clinical notes and facilitate the annotations for various purposes. For example, the multilevel structure can contain the hierarchy and relations of the observed tumor, differentiation, gross type, invasion, size, and other characteristics, while the narrow schema cannot include this information. This rich information can be extracted through the hierarchical schema and can be facilitated for answering a variety of research questions. Therefore, a hierarchical annotation schema is more desirable for clinical research [17-20].

### Comparison With Prior Work

One of the attempts to standardize diverse EHR formats into CDM is the Sentinel project. Sentinel and its component (ie, Mini-Sentinel) have been developed by the United States Food and Drug Administration (FDA), with the aim to create an active surveillance system for monitoring the safety of medical products [21]. Sentinel is a US domestic data model, and the OMOP-CDM was used in this study because of its international research network and wide coverage of standardized medical terminology [22].

In the aspects of NLP frameworks, many NLP information extraction and retrieval systems have been developed to process documents in EHRs for use in clinical practice or research. EMERSE is a clinical note searching system developed using Apache Lucene to increase the availability of EHRs and to help clinicians and researchers effectively retrieve information [23]. SemEHR provides a biomedical information extraction and semantic search system for clinical notes, and several case studies have proven the system’s usability [24]. SemEHR facilitates Fast Healthcare Interoperability Resources (FHIR) to represent the clinical semantic concepts extracted from free text. cTAKES and CLAMP are widely used NLP systems that provide serial components for information extraction [25,26]. CREATE is an information retrieval system based on the OMOP-CDM for executing textual cohort selection queries on structured and unstructured data [27]. On the other hand, Sharma et al proposed a phenotyping system with NLP algorithms to extract features from the clinical documents of the OMOP-CDM database [28].

Despite well-performing systems, using the systems is still difficult since the systems require high optimization for the local environment and extensive domain knowledge [29]. Moreover, clinical note extraction and preprocessing are needed separately from the systems. The lack of a user interface limits the systems’ usability and portability. Hence, clinical NLP systems that can be applied to standardized medical databases and provide serial NLP components to enhance research continuity are required for users. In this study, we chose the OMOP-CDM owing to its wide coverage of standardized medical terminology and worldwide distributed research networks.

### Objectives

This study aimed to integrate unstructured clinical textual data with structured data through the framework referred to as Staged Optimization of Curation, Regularization, and Annotation of clinical text (SOCRATex). The proposed framework was designed (1) to define a flexible hierarchical annotation schema containing complex clinical information through efficient chart review, (2) to generate reusable annotations based on user-configurable JavaScript object notation (JSON) architecture, and (3) to construct a clinical text data repository that can be integrated with the standardized structured data.

## Methods

### System Architecture

**Figure 1:**
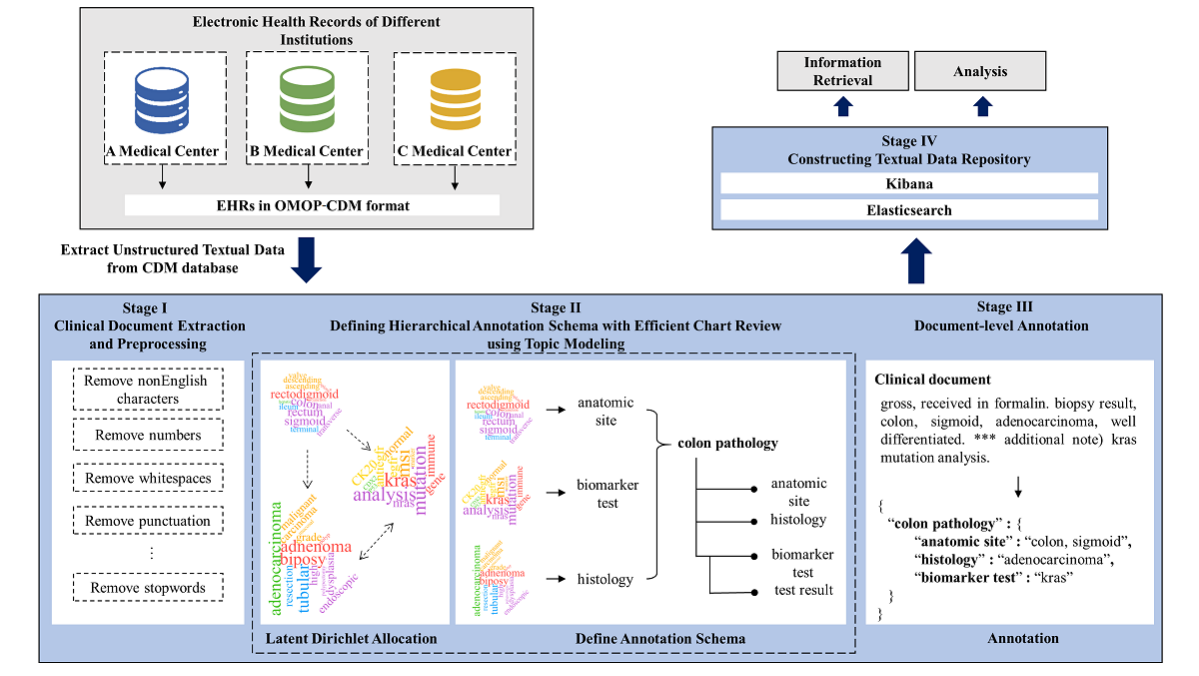
The overall system architecture of Staged Optimization of Curation, Regularization, and Annotation of clinical text (SOCRATex). The system has the following four stages: (1) extracting clinical notes for the target population and preprocessing the data, (2) defining the annotation schema with a hierarchical structure, (3) performing document-level hierarchical annotation using the annotation schema, and (4) indexing annotations for a search engine system. CDM: common data model; EHR: electronic health record; OMOP: Observational Medical Outcomes Partnership.

SOCRATex follows a pipeline-based architecture with the following four stages: (1) extracting clinical notes for the target population and preprocessing the data, (2) defining the annotation schema with a hierarchical structure by referring clustered topics from the clinical notes; (3) performing document-level hierarchical annotation; and (4) constructing a textual data repository with a search engine (Figure 1). All source codes are available online [30].

### Stage 1: Data Extraction and Preprocessing

In the first stage of SOCRATex, the user defines the target population. OHDSI provides an open-source software stack known as ATLAS, which enables users to define complex and transferrable phenotypes of interest based on structured data (ie, diagnosis, medication prescription, medical device use, and laboratory measurements) [31]. The documents in the OMOP-CDM are fully connected to other structured data through patient identifiers. Information regarding note type, language, and encoding system are stored with a fully standardized vocabulary [32]. 

In the NOTE table, foreign keys that can be connected with other tables exist in the CDM (Figures S1 and S2 in Multimedia Appendix 1). NOTE_EVENT_ID is a foreign key identifier of the event (ie, drug exposure, visit, and procedure) during which the note was recorded. NOTE_EVENT_FIELD_CONCEPT_ID is a standardized vocabulary showing which NOTE_EVENT_ID is being referred to. NOTE_TYPE_CONCEPT_ID represents the type, origin, or provenance of the recorded clinical notes. SOCRATex extracts a certain type of clinical document for the target population by using NOTE_TYPE_CONCEPT_ID.

The developed framework provides conventional preprocessing functions, such as eliminating stop words, white spaces, numbers, and punctuations; changing text to lowercase; stemming; and generating a document-term matrix. SOCRATex users can add specific regular expressions or terms to the stop words list.

### Stage 2: Defining the Annotation Schema With a Hierarchical Structure

To define an annotation schema for organizing hierarchical entities of medical documents, researchers with domain knowledge need to review the overall documents of interest thoroughly. By leveraging latent Dirichlet allocation (LDA), which clusters similar words based on the word distributions over documents, SOCRATex automatically identifies topic clusters among documents of interest and provides samples of each cluster to researchers [33,34]. It is assumed that the sampled documents can represent the semantic characteristics of the extracted documents because the topic clusters represent the latent semantics of the documents. Therefore, reviewing the samples can suggest an efficient chart review process rather than reviewing the documents. This reduces redundant labor for reviewing charts of similar content to understand the documents of interest comprehensively.

To calculate the optimal number of topics in LDA, we used perplexity scores, a statistical measure for probabilistic models. Users can decide the best hyperparameters for LDA performance based on the perplexity scores [35-39]. For the interpretation of LDA results, SOCRATex shows both words and documents from their associated topics (Figures S1 and S2 in Multimedia Appendix 2). Based on LDA topics, users can define the annotation schema using the JSON architecture, a machine readable and hierarchical architecture consisting of entity-value pairs.

### Stage 3: Document-Level Annotation With a Defined Schema

Manual annotation is notorious for being an error-prone process. To limit the errors and ensure annotation quality, we applied the JSON schema that can restrict the values and data types of annotation entities [40]. Users need to specify the allowed values of annotation entities using the JSON format. For instance, diameters of observed tumors can be restricted to numeric values. The annotation schema can be distributed to other institutions for generating homogeneous annotations.

### Stage 4: Constructing a Textual Data Repository for Data Exploration and Retrieval

Elastic Stack, a group of open-source products specialized in textual data exploration and retrieval, is used for constructing a textual data repository for the annotations. Elastic Stack is composed of Elasticsearch and Kibana. Elasticsearch is a full-text search and analytics engine for textual data, and Kibana is its visualization dashboard [41]. SOCRATex can index the generated annotations into Elasticsearch, and users can explore their data using Kibana (Figure S1 in Multimedia Appendix 3).

### Validation Using EHRs

We applied SOCRATex against hospital data to validate the usability of the framework. The following three distinctive groups were defined using the OMOP-CDM database of Ajou University School of Medicine [42]: (1) patients who were diagnosed with malignant neoplasms of the colon and rectum between 2014 and 2017, (2) patients who were diagnosed with malignant neoplasms of the thyroid gland and who underwent thyroidectomy between 2014 and 2016, and (3) patients who were diagnosed with major depressive disorder and hospitalized via the emergency department between 2012 and 2018.

From each group of patients, we extracted a specific type of clinical note. Among the patients with colorectal cancer, we extracted their pathology reports with the statement of cancerous lesions of the colon and rectum. Radiology reports of postoperative thyroid ultrasonography were extracted for the patients who underwent thyroidectomy owing to thyroid cancer. Among the patients with major depressive disorder, admission notes were selected and identified with a description of the reason for hospitalization.

**Figure 2:**
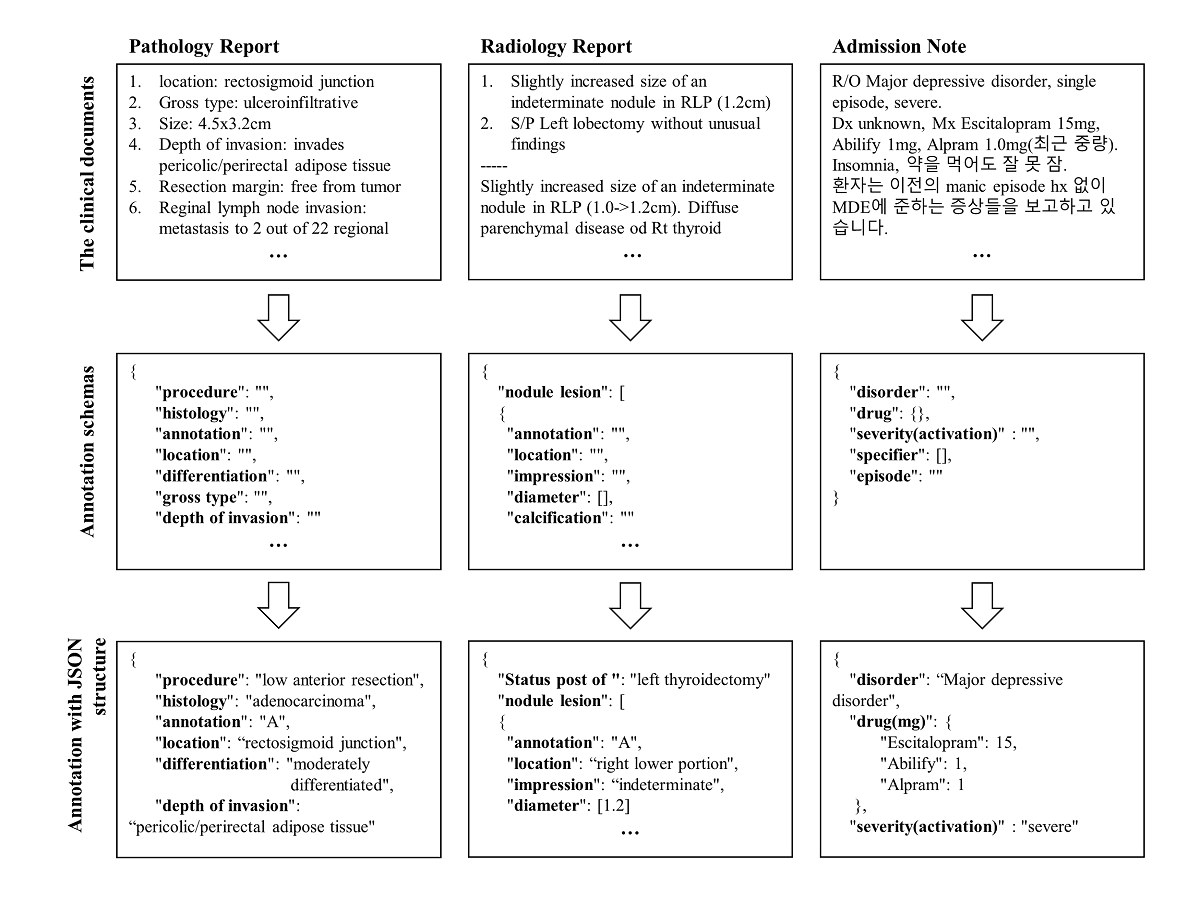
Examples of annotating certain types of clinical documents and their annotation process. Pathology reports have a semistructured format, and radiology reports have a semistructured format with narrative sentences. Admission notes have narrative descriptions in both Korean and English.

Each note type was selected because of its different characteristics (Figure 2). Pathology reports have a semistructured format that is similar to the synoptic pathology reporting form and are primarily written in English [43]. Radiology reports feature a semistructured data format and narrative sentences. Admission notes have narrative descriptions of medical history, disease diagnosis, and medication prescription of the patients. Korean characters were removed and only English characters were included for topic modeling analysis. During the annotation process, we used both languages for accurate annotation. To evaluate the accuracy and efficiency of the SOCRATex annotation process, we compared the annotation process of our system and traditional manual chart review.

Both structured and unstructured textual data were deidentified to protect patient data. The OMOP-CDM per se is a pseudonymized data model that does not allow identifying specific individuals with the data. Hence, it is compliant with pseudonymization of the EU General Data Protection Regulation and Health Insurance Portability and Accountability Act of 1996 (HIPAA) regulations [44,45]. Moreover, the deidentification process in Ajou University Hospital was applied to the data sets to ensure privacy protection (Figure S1 in Multimedia Appendix 4). With the process, patient IDs are encrypted and only the researcher with IRB approval is allowed to receive decryption keys. However, the unstructured textual data can still contain private information. Therefore, a rule-based algorithm was applied to eliminate HIPAA-defined protected health information (PHI) and Korean PHI from the narratives (Tables S1 and S2 in Multimedia Appendix 4). We applied the algorithm by Shin et al that was developed on bilingual clinical documents (ie, Korean and English), was validated on 5000 notes of 33 types, and showed 99.87% precision [46]. The rules for the data set of this study were then optimized and updated.

As proof-of-concept studies, we performed survival analyses to measure mortality rates, cancer recurrence, and hospital readmission using information from both structured clinical data and medical narratives. All-cause mortality, thyroid cancer diagnoses, and hospital readmission information were extracted from structured coded data and defined as outcomes of the analyses. From the annotations, we extracted the following clinical features that were not in structured data: node metastasis, lymphovascular tumor invasion, echogenicity of thyroid nodules, and episodes and specifiers of major depressive disorder. The episodes and specifiers were measured using the Diagnostics and Statistical Manual of Mental Disorder (DSM-5) [47]. Furthermore, we calculated the Korean Thyroid Imaging Reporting and Data System (K-TIRADS) score, a risk stratification of thyroid nodules using the extracted covariates (ie, size, content, and echogenicity of thyroid nodules) [48]. A high K-TIRADS score indicates that the observed thyroid lesions are suspected to be malignant.

In patients diagnosed with colon and rectum cancer, we measured all-cause mortality stratified by node metastasis and lymphovascular invasion. Thyroid cancer recurrence in patients who underwent thyroidectomy was measured with the K-TIRADS score and echogenicity on ultrasonography. Among the patients with major depressive disorder, hospital readmission was measured with specifiers and episodes of major depressive disorder. The *P* value of the log-rank test with Kaplan-Meier curves was measured on each annotation body. We used Cox proportional hazard models to assess and calculate the hazard ratio (HR) between the defined groups. HRs are presented with 95% CIs and *P* values. All *P* values <.05 were considered statistically significant.

To demonstrate external feasibility, we applied SOCRATex to pathology reports from another tertiary hospital’s OMOP-CDM database. This study was approved by the Institutional Review Board at Ajou University Hospital (IRB approval number: AJIRB-MED-MDB-19-579).

## Results

### Stage 1: Defining Patient Groups and Extracting Clinical Documents

Overall, 600 pathology reports from 588 patients with colon and rectum cancer, 308 radiology reports from 220 patients who underwent thyroidectomy, and 147 admission notes from 145 patients with major depressive disorder were included in the study. The characteristics of the patients are shown in Table 1. To compare the cohorts, medical history of the patients was extracted using structured coded data.

Moreover, the information loss and accuracy of clinical note extraction were investigated (Tables S1, S2, and S3 in Multimedia Appendix 1). It showed that data sparsity dropped less than 1% in pathology and radiology reports and 4% in admission notes despite eliminating non-English character removal. The most frequent tokens in documents usually consisted of English characters and a few Korean characters, such as “환자는 (the patient),” “하였다 (did),” and “정신과 (department of psychiatry).”

**Table 1:** Baseline characteristics of the patient groups.

Characteristic			Patients with pathology reports (n=588)		Patients with radiology reports (n=220)		Patients with psychiatric admission notes (n=145)		*P* value
Age (years), mean (SD)			62.65 (12.58)		46.52 (18.69)		49.12 (19.59)		<.001
Female, n (%)			229 (38.9)		176 (80.0)		107 (73.8)		<.001
**General medical history, n (%)**									
	Dementia	6 (1.1)		0 (0.0)		0 (0.0)		.23	
	Gastroesophageal reflux disease	9 (1.5)		8 (3.6)		0 (0.0)		.03	
	Gastrointestinal hemorrhage	31 (5.3)		1 (0.5)		0 (0.0)		<.001	
	Hyperlipidemia	9 (1.5)		11 (5.0)		3 (2.1)		<.001	
	Hypertensive disorder	165 (28.1)		15 (6.8)		2 (1.4)		<.001	
	Diabetes mellitus	84 (14.3)		18 (8.2)		0 (0.0)		<.001	
	Renal impairment	22 (3.7)		3 (1.4)		0 (0.0)		.01	
	Liver lesion	30 (5.1)		1 (0.5)		0 (0.0)		<.001	
**Cardiovascular disease, n (%)**									
	Atrial fibrillation	11 (1.9)		0 (0.0)		1 (0.7)		.08	
	Cerebrovascular disease	6 (1.0)		1 (0.5)		0 (0.0)		.64	
	Coronary arteriosclerosis	10 (1.7)		3 (1.4)		0 (0.0)		.34	
	Heart disease	39 (6.6)		8 (3.6)		1 (0.7)		.008	
	Heart failure	7 (1.2)		2 (0.9)		0 (0.0)		.45	
	Ischemic heart disease	16 (2.7)		2 (0.9)		0 (0.0)		.048	
	Peripheral vascular disease	10 (1.7)		3 (1.4)		1 (0.7)		.86	

### Stage 2: Defining the Annotation Schema With a Hierarchical Structure

The optimal number of topics for pathology reports was determined to be 5, whereas the optimal number of both radiology reports and admission notes was 4 (Table S1 in Multimedia Appendix 2).

**Figure 3:**
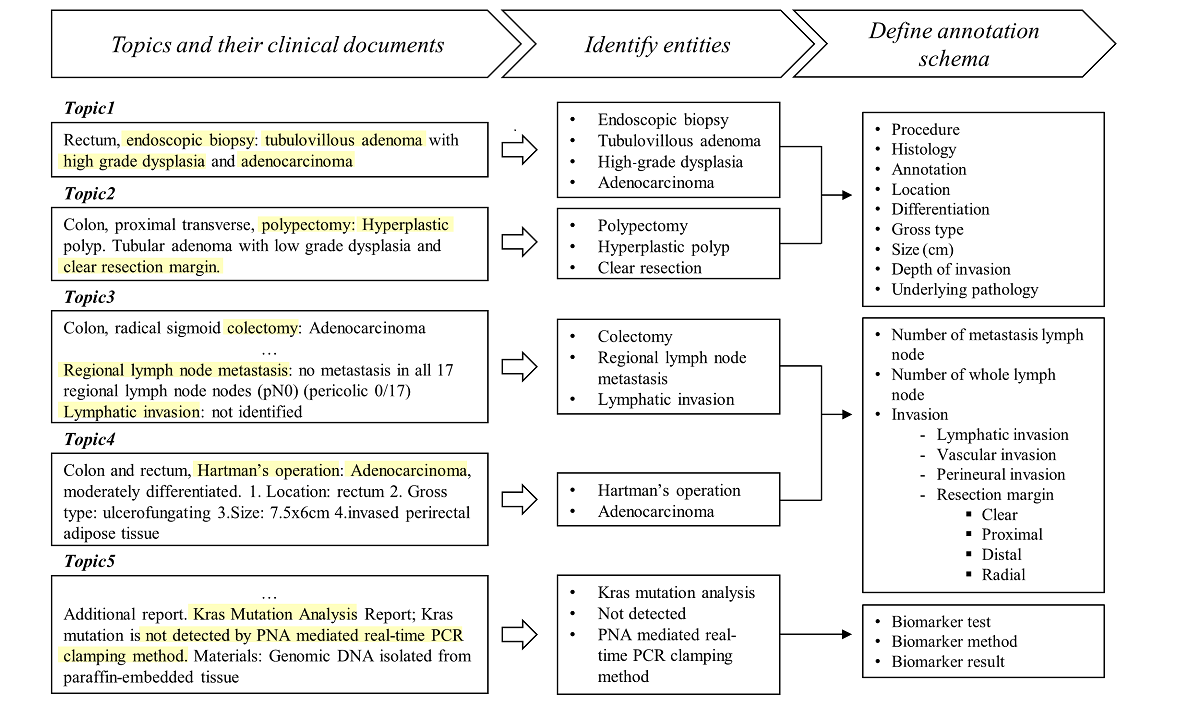
Defining a hierarchical annotation schema of pathology reports, which describes lesions of colon and rectum cancer. The process had the following three steps: (1) classifying documents using clustered topics from the latent Dirichlet allocation model, (2) identifying medical entities of interest, and (3) designing the annotation schema. PCR: polymerase chain reaction; PNA: peptide nucleic acid.

We defined a hierarchical schema of pathology reports based on the topics and sample documents (Figure 3). The entities of pathology reports were classified into the following three groups: lesions, lymph nodes, and biomarker tests. Each entity has a multilevel structure, especially the invasion entity, which showed a deep multilevel structure containing the hierarchical information of lymphatic, vascular, and perineural invasion, and resection margin. The annotation schemas of radiology reports and admission notes are shown in Figures S3 and S4 in Multimedia Appendix 2. Overall, 23 entities were defined for pathology reports, 20 entities for radiology reports, and 5 entities for admission notes.

### Stage 3: Document-Level Annotation With a Defined Schema

Document-level annotation was applied on the extracted documents, resulting in the annotation of 1055 clinical documents with the defined schema. A total of 1000 colonoscopy pathology reports from another tertiary hospital database were annotated with the distributed annotation schema (Multimedia Appendix 5). The comparison between SOCRATex annotation and traditional chart review is described in Multimedia Appendix 6. It shows that the mean accuracy of traditional chart review was 0.917 and its mean annotation time was 548 minutes. On the other hand, the mean accuracy of SOCRATex annotation was 0.937 and its mean annotation time was 360 minutes.

### Stage 4: Constructing a Textual Data Repository for Data Exploration and Retrieval

The generated annotations were indexed into Elasticsearch to construct a textual data repository. Table S1 in Multimedia Appendix 3 demonstrates that the admission notes were identified as having the largest tokens (24,319 tokens) and that the radiology reports were identified as having 1006 tokens. The tokens of pathology reports were 3561.

**Figure 4:**
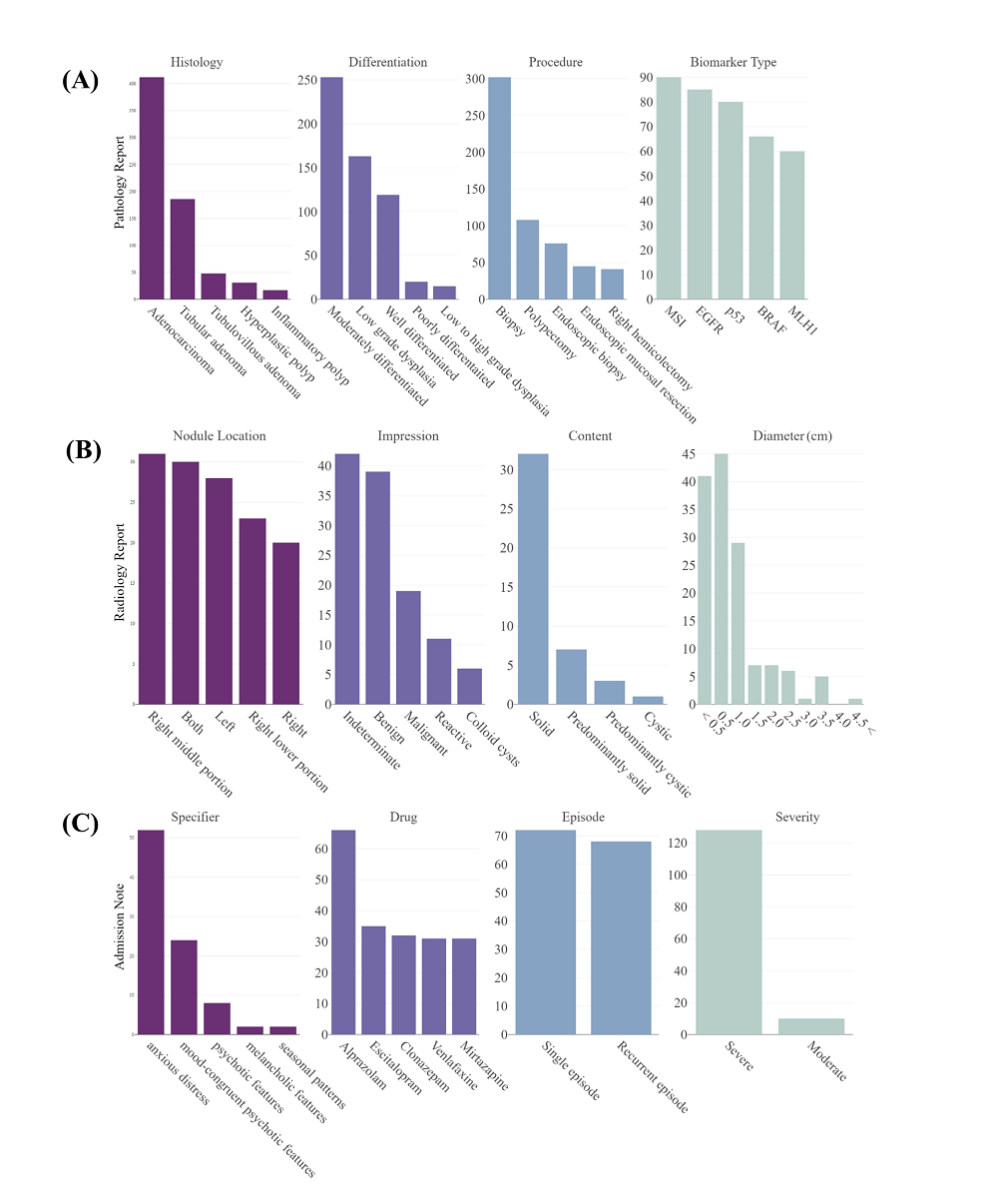
Histograms of annotation entities derived from pathology reports (A), radiology reports (B), and admission notes (C). (A) shows the number of observed histologies, differentiations, procedures, and biomarkers; (B) shows the number of locations, impressions, contents, and diameters of the observed thyroid nodules; and (C) shows the specifiers, episodes, severities, and used medications in major depressive disorder patients.

Using the constructed textual data repository, we explored the entity distributions of the annotations using the Kibana interface (Figure 4). Figure 4A shows the distributions of pathology entities. It shows that adenocarcinoma was the most frequent tumor, which was observed in 412 of 600 documents (68.7%). Tubular and tubulovillous adenomas were the second most frequent tumors, which were observed in 186 (31.0%) and 48 (8.0%) documents, respectively. Among the biomarker tests, the microsatellite instability test was identified as the most frequent biomarker test with 90 (50.3%) occurrences, followed by epidermal growth factor receptor with 85 (47.5%) occurrences. The distributions of radiology entities showed that solid or predominantly solid thyroid nodules were observed in 34 of 148 documents, in which 209 (16.2%) nodules were observed via thyroid ultrasonography (Figure 4B). There were only 4 (2.70%) documents describing cystic or predominantly cystic nodules. Of 144 observed lesions with nodule size, 20 (14.1%) nodules were larger than 2.0 cm and the other 122 (85.9%) nodules were less than 2.0 cm. Using the DSM-5, we identified the severity, episode, and specifier of major depressive disorder from admission notes (Figure 4C). As a result, 52 (35.4%) hospitalized cases and 33 (22.5%) cases were identified as having anxious distress of major depressive disorder and psychotic or mood-congruent psychotic features, respectively. In addition, we identified the medication usage patterns of patients. The most frequently prescribed medication was alprazolam with 66 (22.6%) prescriptions, followed by escitalopram with 35 (11.3%) prescriptions.

**Figure 5:**
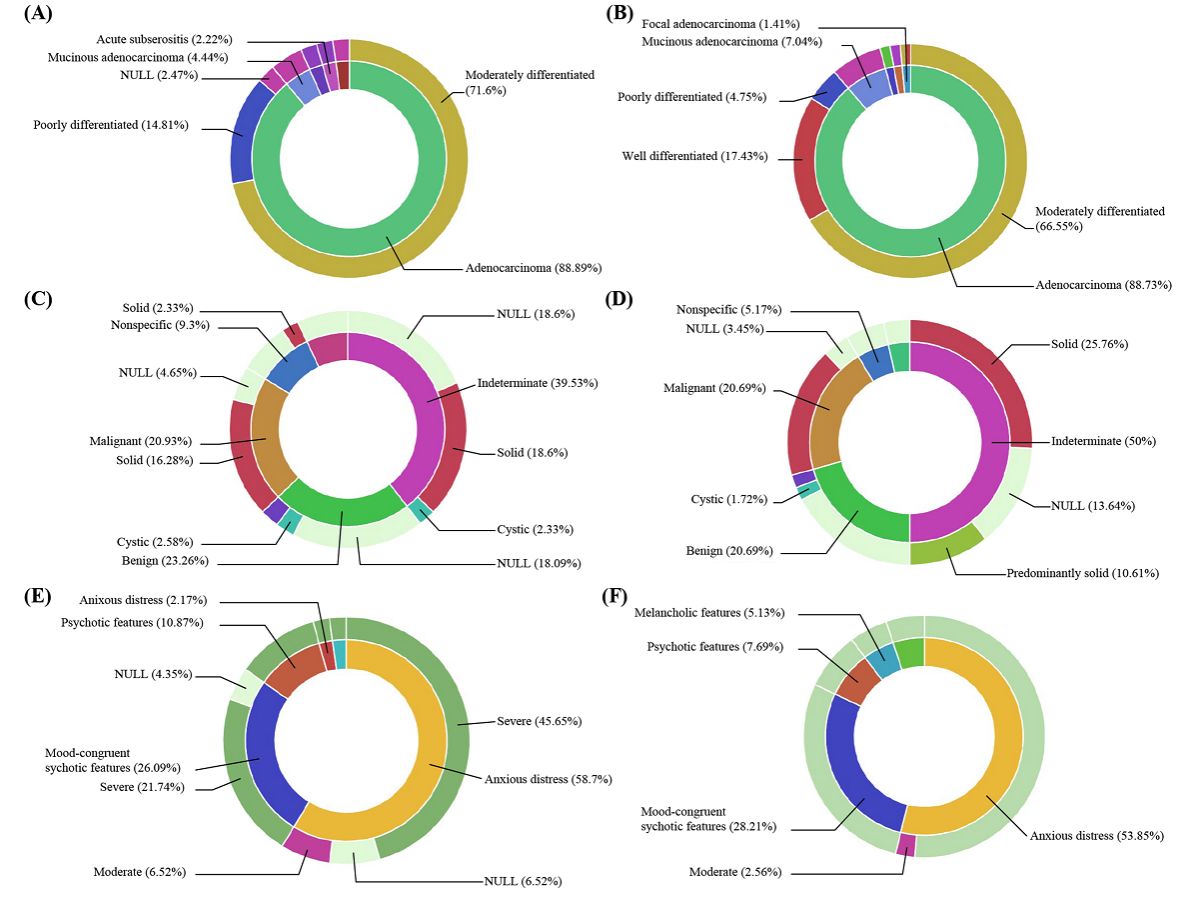
Sunburst plots generated using the Kibana interface. (A) and (B) show the observed histologies and their differentiation from pathology reports. (A) shows the results of lymph node–positive cases, and (B) shows the results of lymph node–negative cases. (C) and (D) are observed from radiology reports. Each of the plots indicates the left and right thyroid in order. (E) and (F) show the disease specifier and its severity from the admission notes. (E) shows the results of single-episode patients, and (F) shows the results of multiple-episode patients.

Hierarchical annotations can show further relationships between the entities. Figure 5 describes the hierarchical relations of the entities. Using Kibana queries, we classified each annotation body into two categories. First, the observed tumors and the differentiation from pathology report findings are described (Figure 5A and 5B). Both are distinguished by lymph node positivity. Among moderately differentiated adenocarcinomas, 29 (71.6%) lymph node–positive cases and 45 (66.6%) lymph node–negative cases were observed. Second, frequent differentiation of adenocarcinoma differed according to lymph node involvement as follows: 6 (14.8%) poorly differentiated in lymph node–positive cases and 11 (17.4%) well differentiated in lymph node–negative cases. Additionally, relations of thyroid nodule types and contents by anatomic locations are described in Figure 5C and 5D. The results show that solid nodules with malignancy were observed in 7 (16.3%) cases in the left thyroid and 10 (14.1%) cases in the right thyroid. On the contrary, benign cystic thyroid nodules were observed in only 2 (2.6%) cases in the left thyroid and 2 (1.7%) cases in the right thyroid. Third, the specifiers and severities of major depressive disorder were identified (Figure 5E and 5F). The results were divided according to single or recurrent episodes of major depressive disorder. Among the patients with single episodes of the disease, 21 (45.7%) cases were identified as involving severe major depressive disorder with an anxious distress specifier. Additionally, 20 (51.3%) cases of multiple episodes were identified as involving an anxious distress specifier with severe symptoms.

The annotation results of the other tertiary hospital database are described in Multimedia Appendix 5. Tubular adenoma observed at the sigmoid colon was the most frequent histology with 131 cases among 720 observed lesions (20.1%), and hyperplastic polyps represented the second most frequent histology in the sigmoid colon with 76 (11.7%) cases.

### Association of Features From Clinical Notes and Structured Data

**Figure 6:**
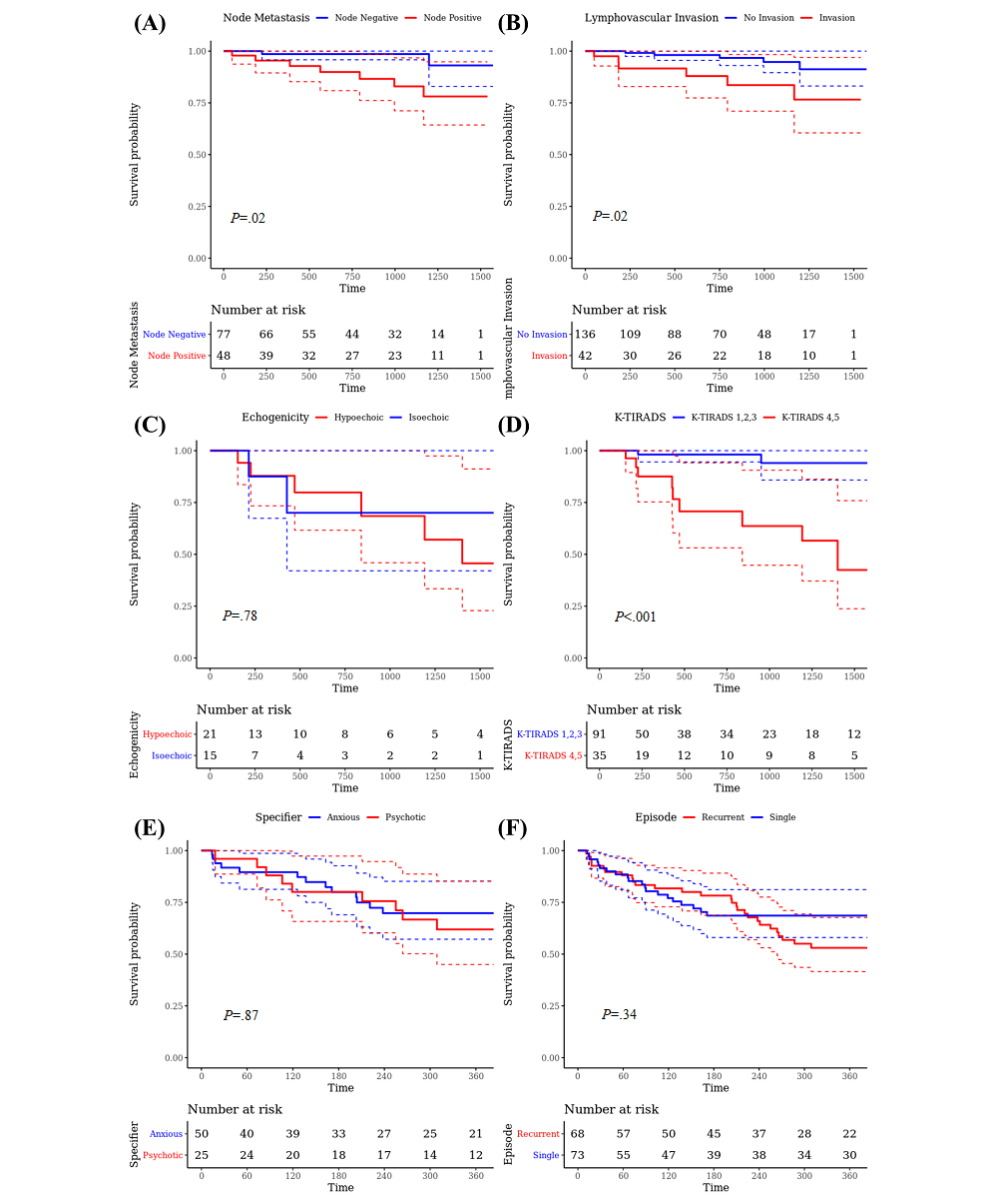
Kaplan-Meier curves with P values of the log-rank test. Survival analyses were performed. (A) and (B) measure 5-year mortality rates of patients with colorectal cancer by node metastasis and lymphovascular tumor invasion, respectively. (C) and (D) measure thyroid cancer recurrence by echogenicity of thyroid nodules and K-TIRADS scores, respectively. (E) and (F) measure 30-day readmission of patients with major depressive disorder by disease specifiers and episodes, respectively. K-TIRADS: Korean Thyroid Imaging Reporting and Data System.

For patients diagnosed with malignant neoplasm of the colon and rectum, 5-year survival analyses were performed (Figure 6A and 6B). The analyses measured mortality rate according to node metastasis and lymphovascular tumor invasion. We found that patients with lymph node involvement had significantly worse survival rates than those without involvement (HR 5.22, 95% CI 1.08-25.22; *P*=.04). Lymphovascular invasion was also associated with significantly higher mortality in patients with colorectal cancer (HR 3.75, 95% CI 1.14-12.32; *P*=.03).

Recurrence risk of thyroid cancer stratified by the echogenicity of thyroid nodules and the K-TIRADS score was measured (Figure 6C and 6D). In our analysis, recurrence of thyroid cancer was not significantly associated with the echogenicity of thyroid nodules (HR 0.80, 95% CI 0.16-3.98; *P*=.78). On the other hand, we found that high K-TIRADS scores (K-TIRADS 3 and 4) were associated with a higher risk of thyroid cancer recurrence compared with low K-TIRADS scores (K-TIRADS 1-3) (HR 12.43, 95% CI 2.73-56.60; *P*<.001).

Among patients with major depressive disorder, we measured 30-day readmission according to disease specifiers and episodes, which were measured based on the DSM-5 (Figure 6E and 6F). The specifiers were classified into anxious distress and psychotic features. Disease episodes were classified into single or recurrent episodes. The results showed that 30-day readmission was not significantly associated with the specifiers of major depressive disorder (HR 1.07, 95% CI 0.50-2.26; *P*=.87). Single or recurrent episodes of major depressive disorder were not significantly associated with 30-day readmission (HR 0.78, 95% CI 0.47-1.29; *P*=.34).

## Discussion

### Principal Findings

The framework succeeded in hierarchically annotating unstructured clinical documents and integrating them into standardized structured data. Through proof-of-concept studies, three different types of clinical documents (ie, pathology reports, radiology reports, and admission notes) were extracted and processed with topic modeling to identify medical concepts. The hierarchical schemas were defined with efficient chart review by sampling documents according to semantic topics. Overall, 1055 documents were manually annotated using the schemas and indexed in the search engine. We attempted multidimensional validation by identifying the characteristics of the hierarchical annotations and by performing survival analyses with integrated data of structured and unstructured textual information. The following were identified through validation: (1) the association of node positivity with mortality in patients with colorectal cancer, (2) the association of the K-TIRADS score with thyroid cancer mortality, and (3) medication usage patterns according to depression episodes.

SOCRATex uses flexible annotation schemas for clinical text annotation that can include complex information in free-text documents (Multimedia Appendix 7). The narrow annotation schema can only extract the entities of disease, treatment, and test [15,16]. These simple entities are effective to annotate and train the model, but difficult to explain their relationships. On the contrary, the annotation schema on pathology reports successfully contained the relationships among tumor type, dimension, location, and invasion. Consequently, we identified that more than 42% (253/588) of colorectal cancer patients had moderately differentiated adenocarcinoma and underwent a microsatellite instability test. In the radiology reports, 23% (34/148) of thyroid nodules were identified as having solid content. The hierarchical schema of admission notes identified medication usage patterns by disease episodes, showing that alprazolam and escitalopram were the most frequently prescribed medications in both patient groups.

Through proof-of-concept studies, we demonstrated that the generated hierarchical annotations could be used in various settings of clinical research. The survival analyses of patients with colorectal cancer showed that node positivity and lymphovascular invasion were significantly associated with a higher mortality rate, which is consistent with the findings of previous studies [49,50]. The analyses of radiology reports found that higher K-TIRADS scores were significantly associated with the recurrence of thyroid cancer, which is consistent with previous reports [48].

### Limitations

This study has several limitations that can direct future research. First, interesting clinical implications were not determined from our proof-of-concept studies. To discover novel medical evidence, a sophisticated study design is required. However, our aim here was to demonstrate that the generated textual data repository could be used for clinical research. Second, the feasibility of the framework in the distributed research network was not fully validated. Still, we distributed the annotation schema of pathology reports to the other institution and were able to annotate 1000 colonoscopy pathology reports. Third, the defined annotation schema was not systemically evaluated. Three annotation schemas were defined with domain experts according to their related clinical domains. However, systematic validation of the schemas is still required. Moreover, the applicability of FHIR standards in the system of this study will be investigated to test its extensibility.

Although the generated annotations can be reused for clinical analyses of various purposes, the initial manual annotation of documents is still a time-consuming and costly process. In future work, state-of-the-art algorithms, such as BERT, XLNet, and GPT-3, could be applied to automatic information extraction processes to reduce the annotation burden and cost [51-53].

### Conclusions

We propose a clinical text processing framework to generate flexible hierarchical annotations and integrate them with the standardized structured data of the OMOP-CDM. The proof-of-concept studies demonstrated that the generated annotations were integrated with the structured data and were successfully used for various clinical research approaches with efficient chart review processes. The conformance with CDM allows the application of a standard annotation schema to generate homogeneous annotations from different institutions.

## Appendix

Multimedia Appendix 1 Clinical note data extraction, processing, and validation.

Multimedia Appendix 2 Evaluating the latent Dirichlet allocation model performance and defining annotation schemas.

Multimedia Appendix 3 Staged Optimization of Curation, Regularization, and Annotation of clinical text (SOCRATex) annotation and information retrieval system.

Multimedia Appendix 4 Protecting and deidentifying patient information.

Multimedia Appendix 5 Study results from Samsung Medical Center.

Multimedia Appendix 6 Comparison between Staged Optimization of Curation, Regularization, and Annotation of clinical text (SOCRATex) annotation and traditional chart review.

Multimedia Appendix 7 Comparison between Staged Optimization of Curation, Regularization, and Annotation of clinical text (SOCRATex) and other natural language processing systems.

## Abbreviations

CDM: common data model
DSM-5: Diagnostics and Statistical Manual of Mental Disorder
EHR: electronic health record
FHIR: Fast Healthcare Interoperability Resources
HIPAA: Health Insurance Portability and Accountability Act
HR: hazard ratio
JSON: JavaScript object notation
K-TIRADS: Korean Thyroid Imaging Reporting and Data System
LDA: latent Dirichlet allocation
NER: named entity recognition
NLP: natural language processing
OHDSI: Observational Health Data Sciences and Informatics
OMOP: Observational Medical Outcomes Partnership
PHI: protected health information
SOCRATex: Staged Optimization of Curation, Regularization, and Annotation of clinical text
