# Supplementary material for: A Framework (SOCRATex) for Hierarchical Annotation of Unstructured Electronic Health Records and Integration Into a Standardized Medical Database: Development and Usability Study
Source: JMIR Med Inform. 2021 Mar 30;9(3):e23983. doi: 10.2196/23983 (PMC8044740; doi:10.2196/23983)
Supplement: Multimedia Appendix 1 [file medinform_v9i3e23983_app1.docx]

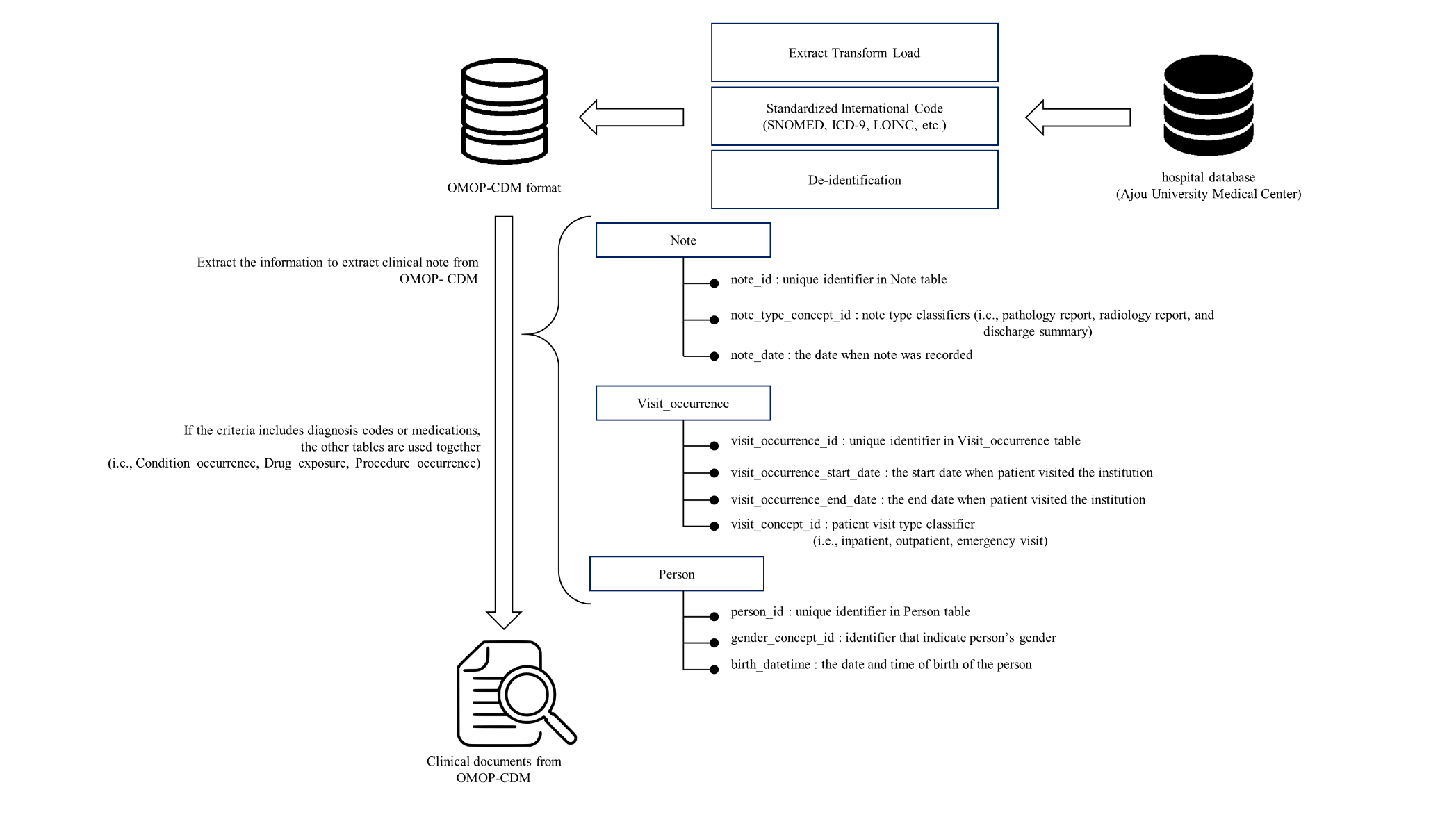


Figure S1. NOTE data extraction and processing in OMOP-CDM


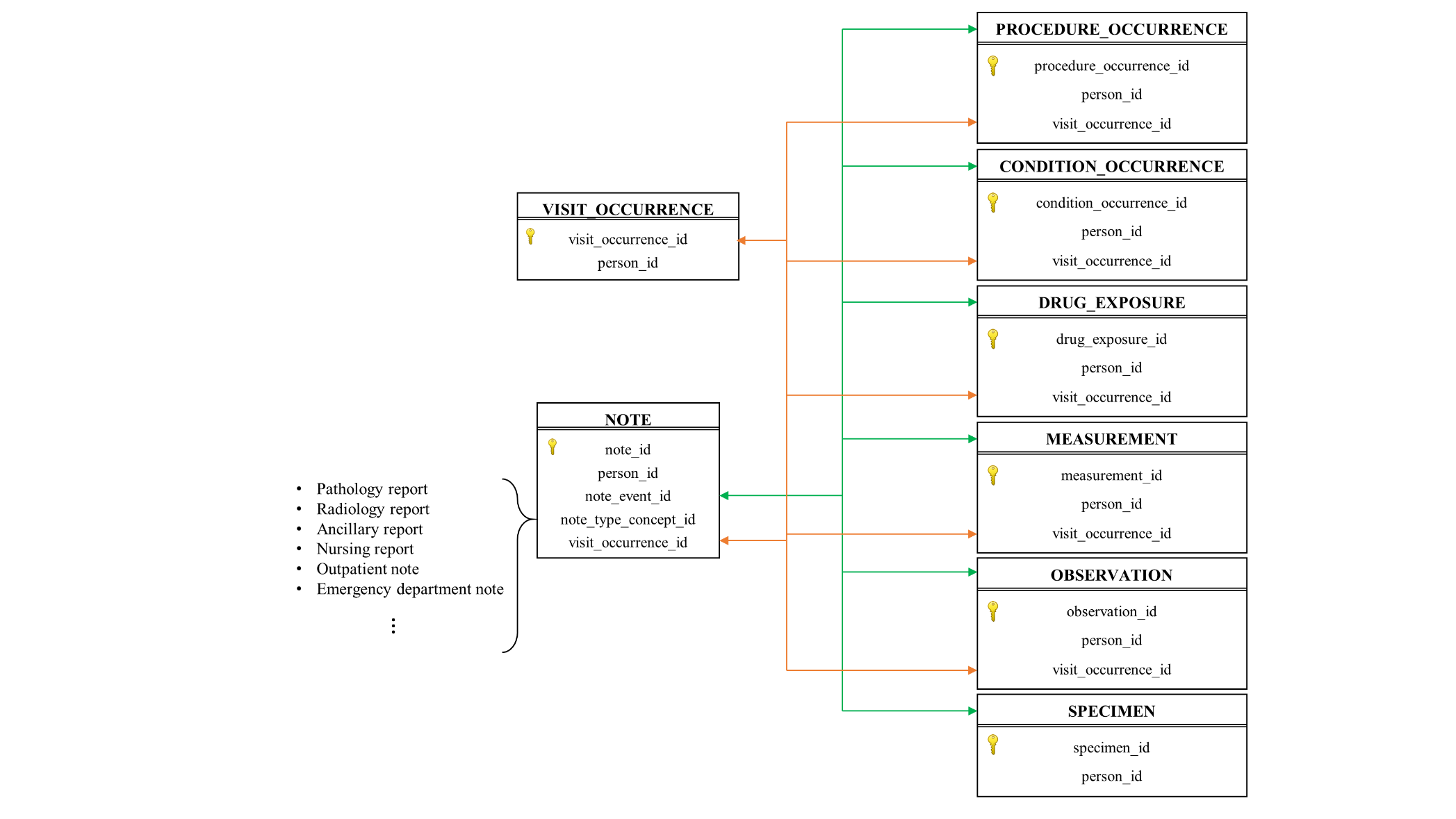


Figure S2. Identifying clinical notes and relevant clinical events in OMOP-CDM

Table S1. Number of tokens and their sparsity according to the inclusion of non-English characters

| **Note type** |  | **Only English characters** | **With non-English characters** |
| --- | --- | --- | --- |
| Pathology | Tokens, n | 1490 | 2521 |
|  | Sparsity (%) | 96 | 97 |
| Radiology | Tokens, n | 434 | 515 |
|  | Sparsity (%) | 94 | 95 |
| Admission | Tokens, n | 1814 | 20179 |
|  | Sparsity (%) | 94 | 98 |

Table S2. Most frequent tokens of note types according to the inclusion of non-English characters

| **Note Type** | **Inclusion of non-English character** | **Tokens** |
| --- | --- | --- |
| Pathology | With only English | colon, specimen, margin, biopsi, resect, anal, verg, one, adenoma, tissue, consist, invas, section, label, low, measur, adenocarcinoma, grade, block, tubular |
|  | With non-English | colon, specimen, margin, biopsi, resect, anal, verg, one, adenoma, tissue, consist, invas, section, label, low, measur, adenocarcinoma, grade, block, tubular |
| Radiology | With only English | thyroid, lesion, lymphadenopathi, clinic, focal, suspici, nodul, portion, right, definit, cervic, bed, left, total, lymph, node, evid, metastasi, calcif, recurr |
|  | With non-English | thyroid, lesion, lymphadenopathi, clinic, focal, suspici, nodul, portion, right, definit, cervic, bed, left, total, lymph, node, evid, metastasi, calcif, recurr |
| Admission | With only English | depress, loss, symptom, disord, mood, reason, visit, episod, major, histori, suicid, anxieti, sever, fhx, tab, insomnia, medic, ideat, month, psychot |
|  | With non-English | 환자는, depress, loss, symptom, disord, mood, reason, visit, major, histori, episod, siocid, 하였다, 환자의, sever, 정신과, fhx, anxiety, tab, 않았다. |

Table S3. Accuracy of psychiatric medication records from clinical note and structured data.

|  | **Clinical note (n)** | **Structured data (n)** | **Accuracy** |
| --- | --- | --- | --- |
| Alprazolam | 66 | 56 | 1.00 |
| Escitalopram | 35 | 37 | 0.95 |
| Clonazepam | 32 | 37 | 0.86 |
| Venlafaxine | 31 | 41 | 0.77 |
| Mirtazapine | 31 | 29 | 1.00 |
| Overall | 195 | 200 | 0.97 |

Table S4. Data dictionary for Stage I in SOCRATex. Through Stage I, note_text column data is converted into word embedded vector matrix and can be inserted into Stage II.

| **Field** | **Type** | **Description** |
| --- | --- | --- |
| note_id | Integer | A unique identifier for each note. |
| person_id | Integer | A foreign key identifier to the Person about whom the Note was recorded. The demographic details of that Person are stored in PERSON table. |
| note_date | Date | The date the note was recorded. |
| note_type_concept_id | Integer | A foreign key to the predefined Concept in the Standardized Vocabularies reflecting the type, origin, or provenance of the Note. These belong to the ‘Note Type’ vocabulary. |
| note_text | Varchar(max) | The content of the Note. |
