# Supplementary material for: A Framework (SOCRATex) for Hierarchical Annotation of Unstructured Electronic Health Records and Integration Into a Standardized Medical Database: Development and Usability Study
Source: JMIR Med Inform. 2021 Mar 30;9(3):e23983. doi: 10.2196/23983 (PMC8044740; doi:10.2196/23983)
Supplement: Multimedia Appendix 2 [file medinform_v9i3e23983_app2.docx]

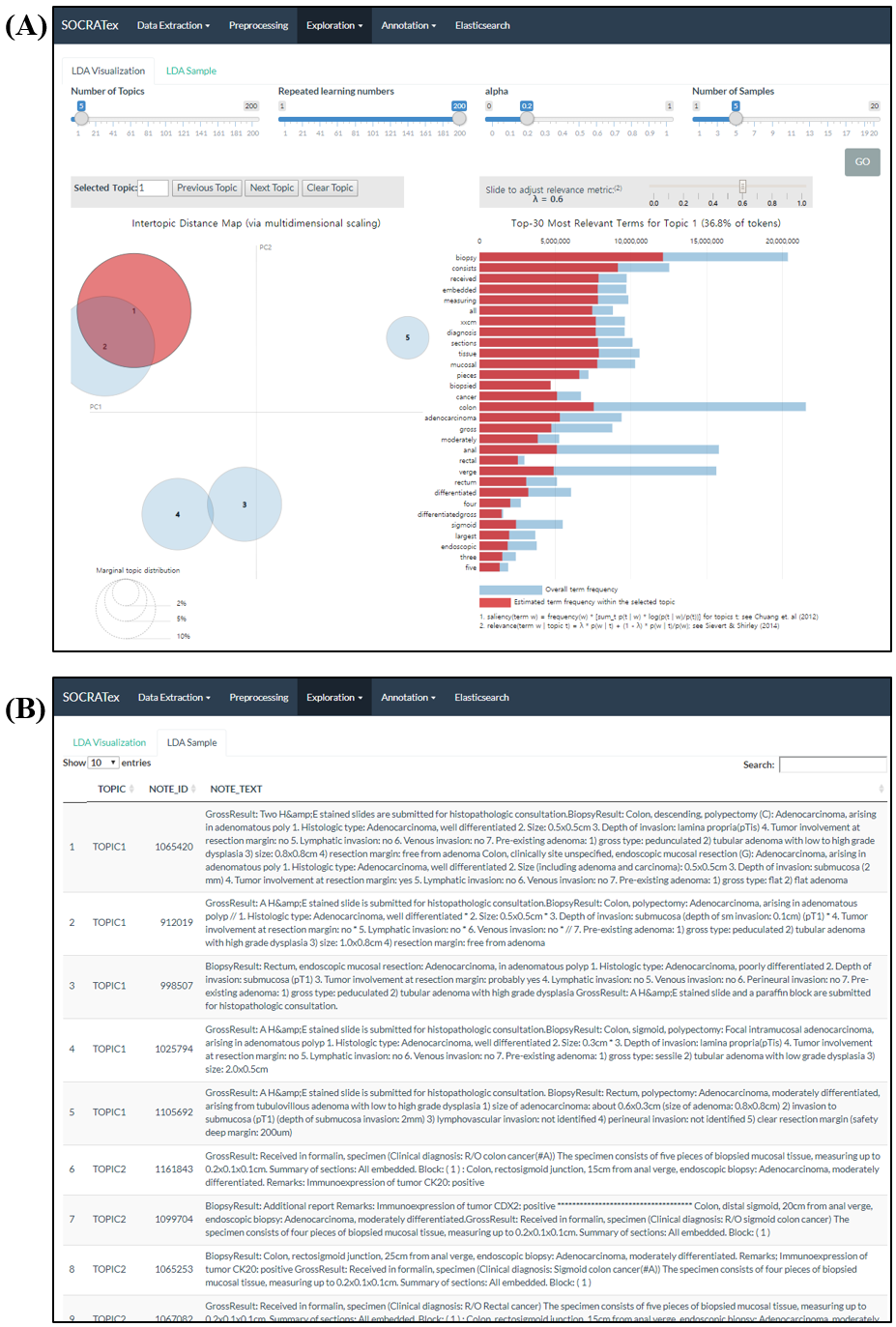


Figure S1. The example views of SOCRATex performing Latent Dirichlet Allocation (LDA) analysis. (A) is showing the interactive LDA analysis results and (B) is showing the sample documents belongs to the topics.


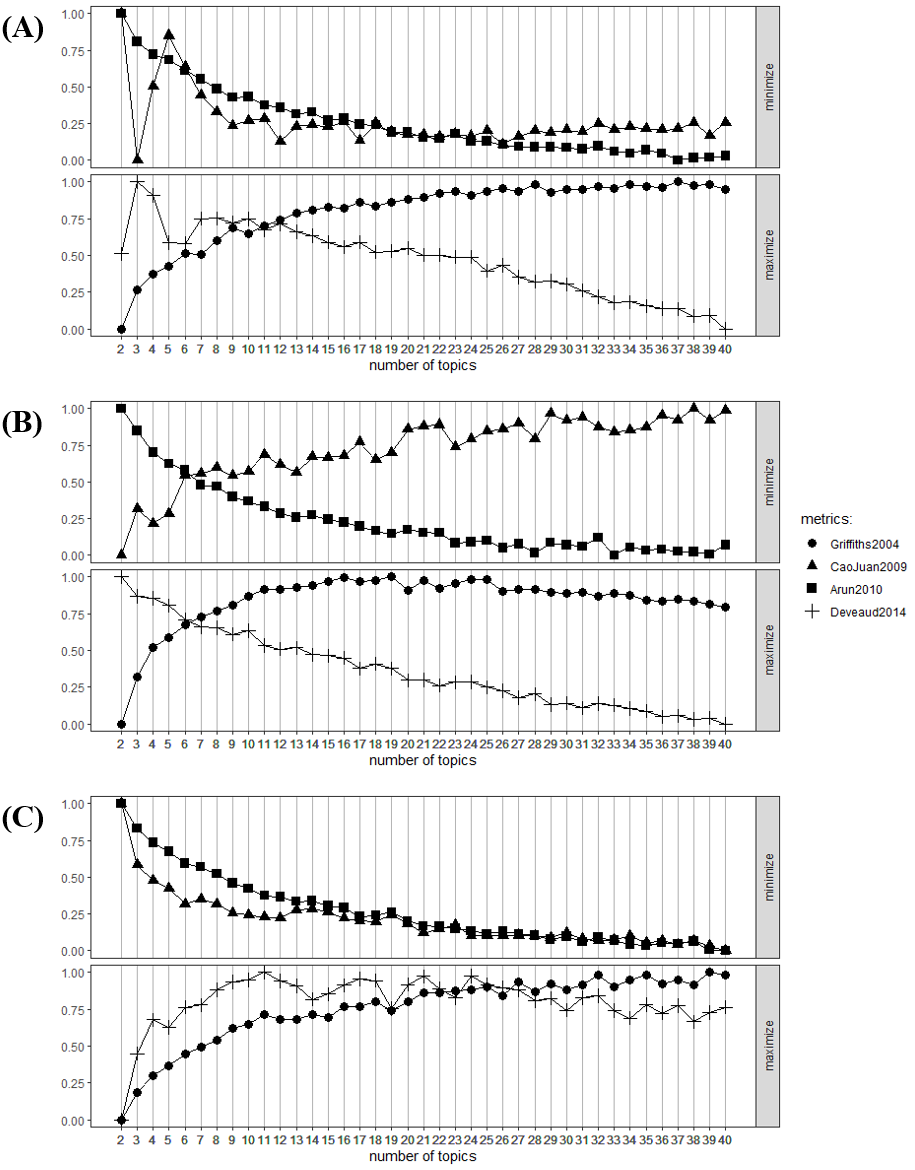


Figure S2. The perplexity graphs which indicate the optimal number of LDA topics of each corpus. (A) is calculated from the pathology reports and (B) is calculated from the radiology reports. (C) is calculated from the admission notes.


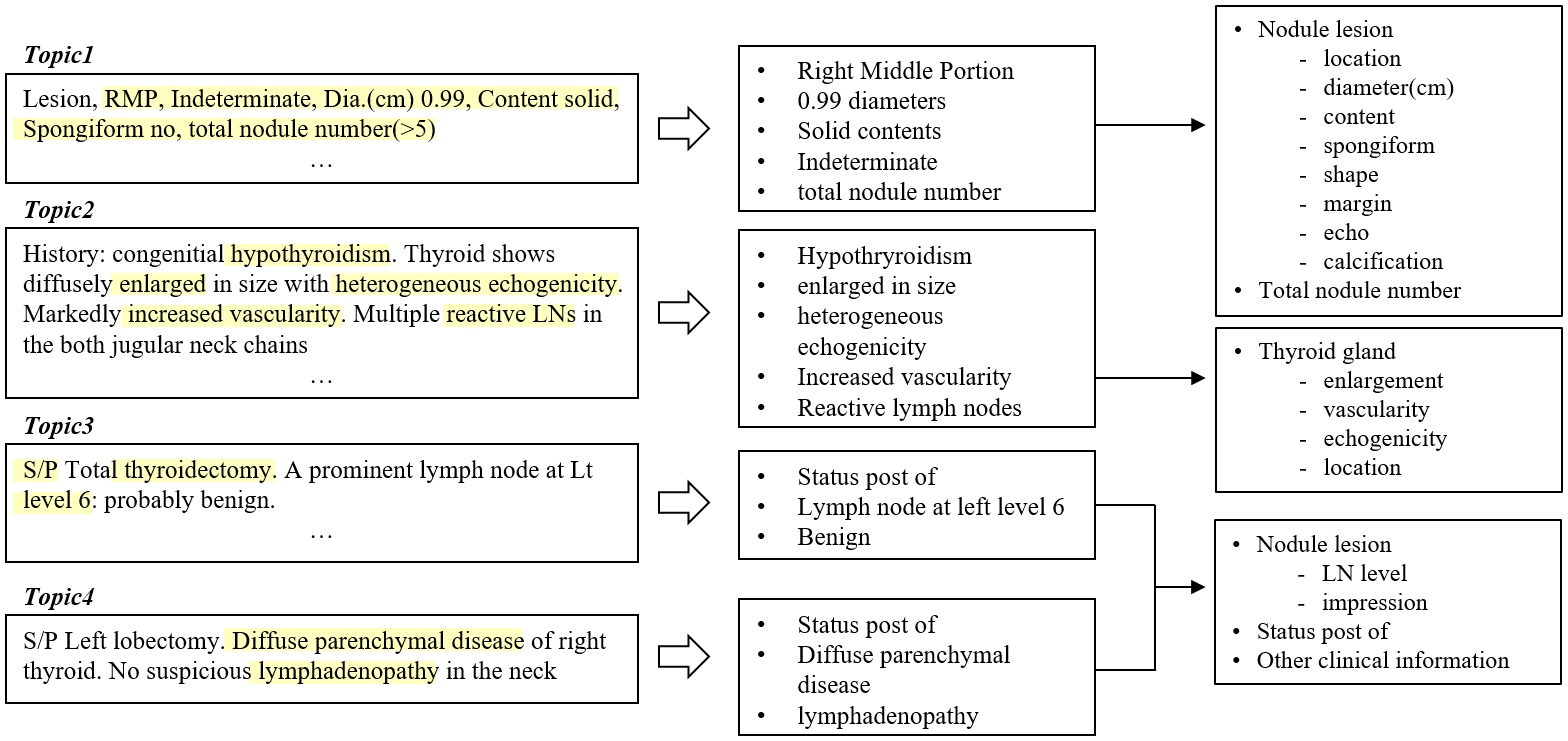


Figure S3. The process of defining data structure of thyroid ultrasonography related radiology reports


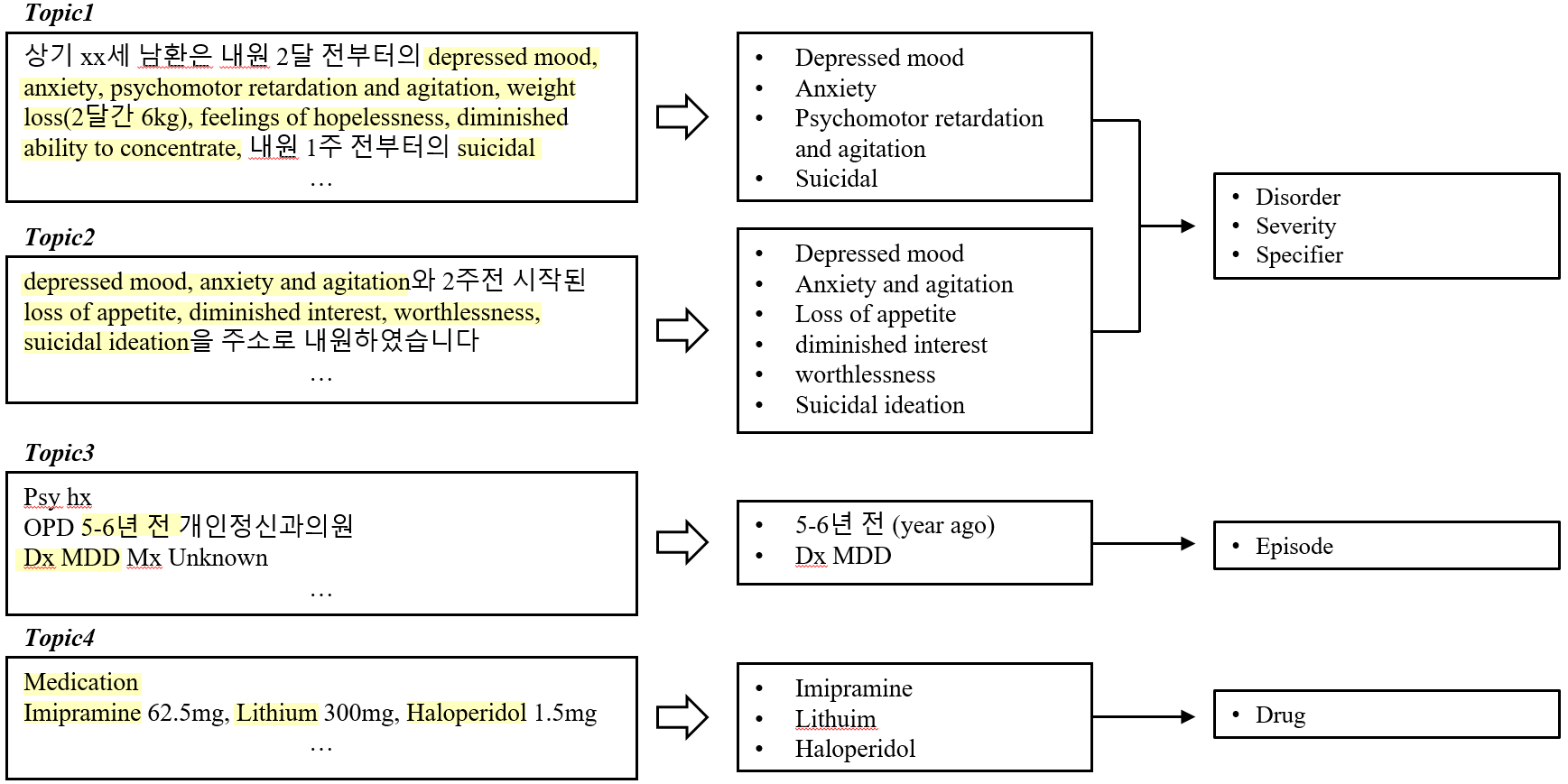


Figure S4. The process of defining data structure of major depressive disorder diagnosed patients’ admission notes.

Table S1. The result of Latent Dirichlet Allocation of each corpus. The topic annotation is labeled by the authors.

| **Note** | **Topic** | **Terms** | **Annotation** |
| --- | --- | --- | --- |
| Pathology reports | Topic1 | biopsy, all, consists, xxcm, embedded, mucosal, received, measuring, diagnosis, sections, tissue, pieces, labelled, gross, biopsied, adenocarcinoma, cancer, differentiated, colon, moderately, rectal, verge, rectum, anal, four, sigmoid, endoscopic, largest, five, one | Malignant, biopsy |
|  | Topic2 | anal, verge, colon, one, tubular, adenoma, low, grade, dysplasia, biopsy, transverse, polypectomy, containers, each, ascending, identified, consists, two, polyp, largest, sigmoid, descending, polypoid, hyperplastic, mucosal, proximal, endoscopic, polyps, xxcm, three | Benign, biopsy |
|  | Topic3 | margin, resection, mass, lymph, invasion, node, regional, xcm, metastasis, distal, apart, len, pericolic, identified, proximal, carcinoma, circumference, nodes, fresh, some, free, illdefined, state, cut, instability, test, msimicrosatellite, bat, invades, iple | Lymph node invasion, surgery |
|  | Topic4 | invasion, adenoma, resection, margin, submitted, consu, ation, hampe, grade, histopathologic, stained, size, adenocarcinoma, dysplasia, high, tumor, tublovillous, type, depth, low, biopsy, gross, well, tubular, labelled, differentiated, polypectomy, colon, endoscopic, whitish | Cancer, surgery |
|  | Topic5 | kras, mutation, analysis, dna, realtime, clamping, pcr, codon, comments, antiegfr, rapy, msi, using, genomic, isolated, mediated, paraffinembedded, target, cetuximab, panitumumab, marker, pnamediated, materials, erlotinib, gefitinib, kinase, tyrosine, inhibitor, pna, additional | Gene mutation analysis |
| Radiology reports | Topic1 | bed, definite, suspicious, focal, lesion, lymphadenopathy, lymph, node, recurrence, tumor, metastasis, evidence, local, thyroidectomy, cervical, neckno, lobectomy, findings, unusual, left, right, necksp, neck, lateral, operation, remnant, prominent, probably, benignno, suture | Unusual findings |
|  | Topic2 | nodule, change, benign, thyroid, nodules, indeterminate, small, tiny, size, several, interval, since, colloid, cysts, cyst, two, neck, benignlooking, rec, compared, previous, rmp, cystic, slightly, rlp, llp, looking, lymphadenopathy, level, multiple | Lesion impression |
|  | Topic3 | thyroid, gland, diffuse, disease, glands, reactive, lns, remarkable, parenchymal, neck, normal, parotid, bilateral, enlarged, echogenecity, vascularity, size, echogenicity, submandibular, lesion, heterogenous, increased, enlargement, area, heterogeneous, focal, finding, level, mild, hypothyroidism | Thyroid gland |
|  | Topic4 | portion, rim, calcification, middle, calcif, dia, upper, homo, right, lower, hypo, left, iso, ovoidround, ill, solid, rmp, macro, hetero, micro, lmp, smooth, hyperechoic, margin, hyper, spongiform, well, macrocalcification, isoechoic, content | Nodule lesion description |
| Admission notes | Topic1 | depress, symptom, reason, visit, medic, mood, histori, month, loss, insomnia, suicid, for, opd, durat, person, ideat, assessment, unknown, disord, plan, brief, interest, psi, anxiety, lorazepam, present, escitalopram, fhx, mse | disorder |
|  | Topic2 | check, loss, axis, depress, speech, presev, thought, suicide, content, gab, impair, mood, insight, not, problem, process, attent, characterist, remot, ideat, concentration, abstract, emotion, sensorium, grasp, capac, spontan, test, behavior, conscious | severity |
|  | Topic3 | depress, psychot, episode, disord, major, venexor, feature, anxiety, stilinox sever, mood, distress, Lexapro, anxious, Xanax, symptom, fhx, rivotril, loss, alpram, moodcongru, reason, visit, singl, delus, panic, histori, intact, phx, abilifi | Specifier, episode |
|  | Topic4 | tab, pain, cap, seroxat, depend, trittico, without, etravil, bid, physiology, zoloft, xanax, buspar, mgml, somat, rivotril, concern, htn, penid, curan, disord, drink, anaphylax, valium, drug, alcohol, mdd, remiss, somatoform, depakin | drug |
