# Supplementary material for: A Framework (SOCRATex) for Hierarchical Annotation of Unstructured Electronic Health Records and Integration Into a Standardized Medical Database: Development and Usability Study
Source: JMIR Med Inform. 2021 Mar 30;9(3):e23983. doi: 10.2196/23983 (PMC8044740; doi:10.2196/23983)
Supplement: Multimedia Appendix 3 [file medinform_v9i3e23983_app3.docx]

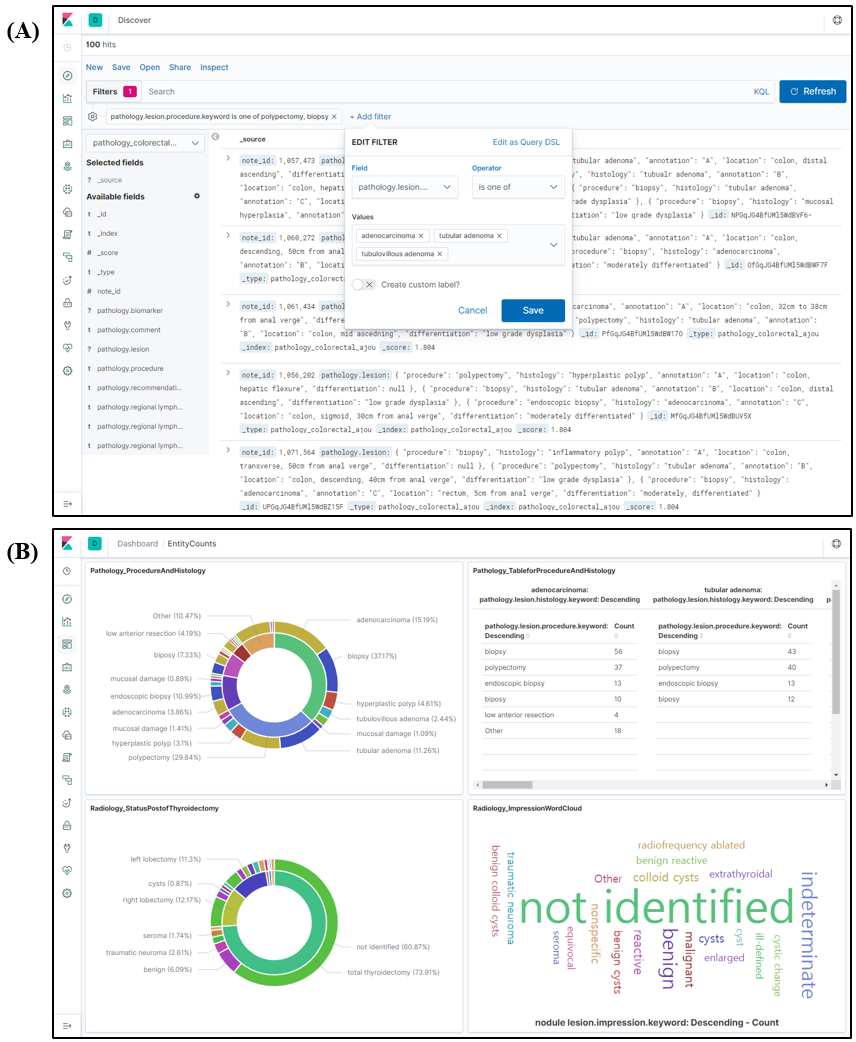


Figure S1. The Kibana interface of visualizing the generated annotation. (A) is exploring with Kibana query and (B) is visualizing the characteristics of the corpus.


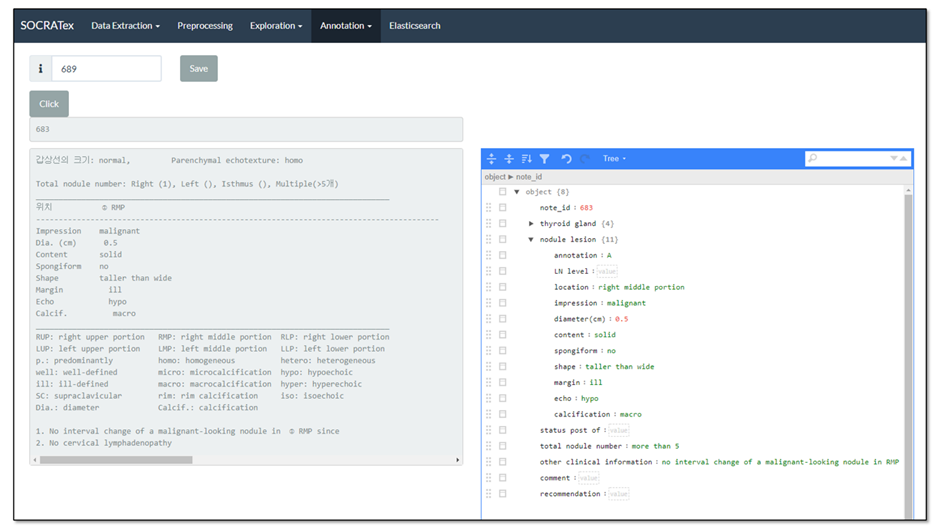


Figure S2. Annotating neck thyroid ultrasound report using SORCRATex system.

Table S1. The number of documents, tokens, and their storage in Elasticsearch of each corpus.

| Report Type | Documents | Tokens | Storage, KB |
| --- | --- | --- | --- |
| Pathology reports | 600 | 3,561 | 396.8 |
| Radiology reports | 300 | 1,006 | 176.6 |
| Admission notes | 147 | 24,319 | 73.1 |
