# Supplementary material for: A Framework (SOCRATex) for Hierarchical Annotation of Unstructured Electronic Health Records and Integration Into a Standardized Medical Database: Development and Usability Study
Source: JMIR Med Inform. 2021 Mar 30;9(3):e23983. doi: 10.2196/23983 (PMC8044740; doi:10.2196/23983)
Supplement: Multimedia Appendix 4 [file medinform_v9i3e23983_app4.docx]

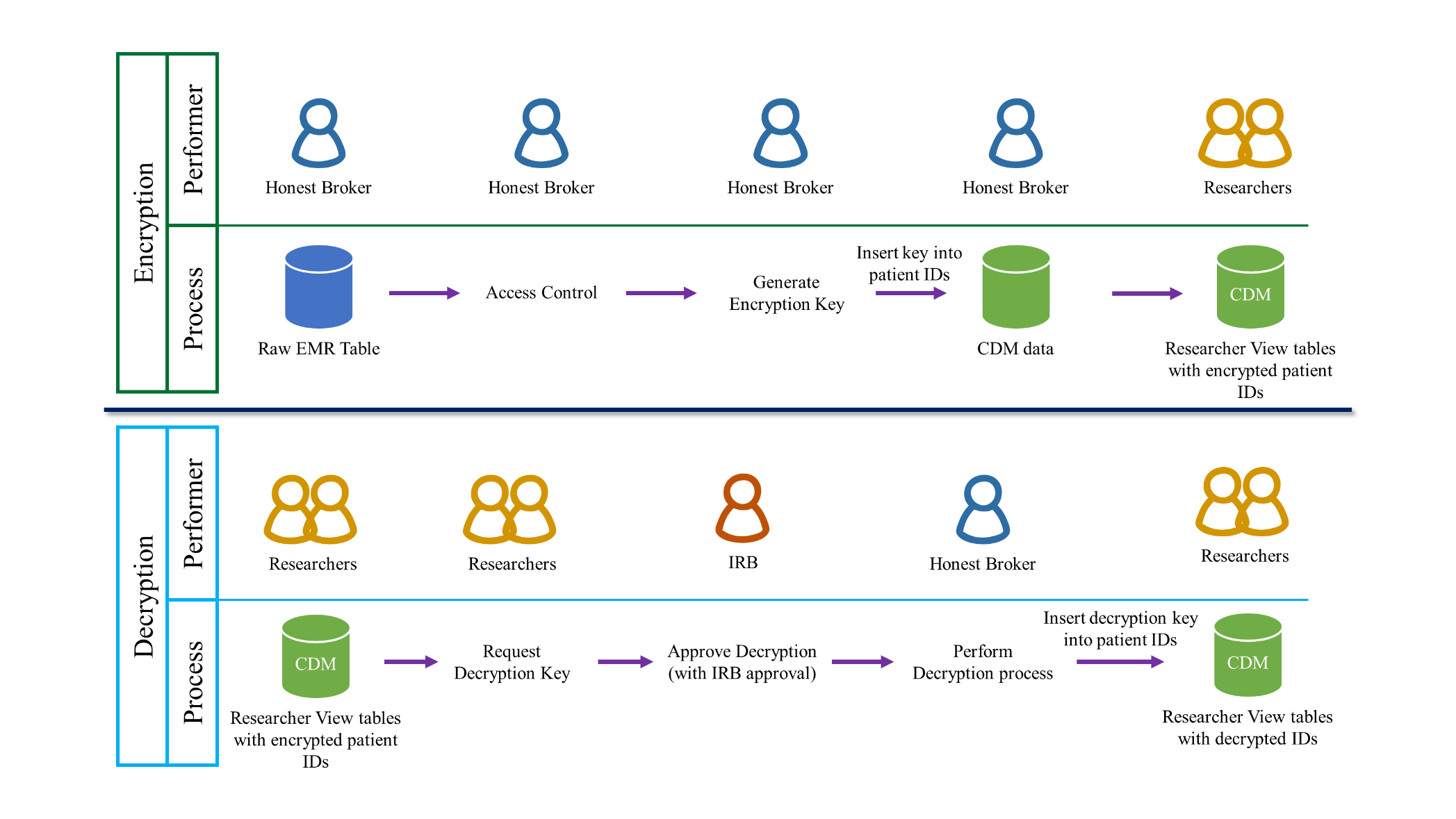


Figure S1. Encryption and decryption process in Ajou University Hospital (i.e., Honest Broker).

Table S1. HIPAA defined PHI existence in notes used in the study

| **Protected Health Information** | **Exist in notes** |
| --- | --- |
| Names of patients and providers | Y |
| All geographical identifiers and entities that indicates recognizable locations or institutions | Y |
| Dates (other than year) directly related to an individual | Y |
| Phone Numbers | N |
| Fax numbers | N |
| Email addresses | N |
| Social Security numbers | N |
| Medical record numbers | Y |
| Health insurance beneficiary numbers | N |
| Account numbers | N |
| Certificate/license numbers | N |
| Vehicle identifiers | N |
| Device identifiers | N |
| Web Uniform Resource Locators (URLs) | N |
| Internet Protocol (IP) address numbers | N |
| Biometric identifiers, including finger, retinal and voice prints | N |
| Full face photographic images and any comparable images | N |
| Any other unique identifying number, characteristic, or code except the unique code assigned by the investigator to code the data | N |

Table S2. Previously defined regular expression rules to detect Korean and HIPAA PHI and added rules

| **Patterns of identifiers** | **Previously Defined Rules** | **Added Rules** |
| --- | --- | --- |
| **Names** |  |  |
| Name of patient | (labelled as \".+\")\|(labelled as \"\") | Exact match using list of publicly available Korean names |
| Relatives of patient or name of physician | //[a-zA-Z]+\| <[/?a-zA-Z]+> | Exact match using list of publicly available Korean names |
| **Geographical identifiers** | (labelled as \".+\")\|(labelled as \"\") | ((강원\|경기\|경상\|전라\|충청).*(시\|군\|구)\s)\|((울산\|인천\|대구\|부산\|서울\|대전\|광주).*(구\|면\|군)\s)\|(세종.*(동\|읍\|면)\s)\|(제주.*(시)\s) |
| **Patient IDs** | [[a-zA-Z][0-9]+-[0-9]+]\| ([a-zA-Z][0-9]+-[0-9]+.+) | (등록번호\|등록 번호\|환자번호\|환자 번호\|환자 정보\| ID\|id\|IDNO\|idno\|NO\|No\|Baby\|baby\|기증자\|수혜자\| Donor\|donor\|Recipient\|recepient)\D{0,5}\d{1,10} |
| **Dates** | [0-9]+/[0-9]+/[0-9]+ | (생.*일\|birth\|BIRTH\|Birth).{1,3}\d{2,4}\d{1,2}\d{1,2} |
|  | [0-9]+년.[0-9]+월.[0-9]+일 | 출.*생.{1,3}\d{2,4}\d{1,2}\d{1,2} |
