# Supplementary material for: A Framework (SOCRATex) for Hierarchical Annotation of Unstructured Electronic Health Records and Integration Into a Standardized Medical Database: Development and Usability Study
Source: JMIR Med Inform. 2021 Mar 30;9(3):e23983. doi: 10.2196/23983 (PMC8044740; doi:10.2196/23983)

**Multimedia Appendix 5**

Pathology reports of Samsung Medical Center (SMC) were used for the validation of the proposed application and annotation schema generated from Ajou University School of Medicine (AUSOM) pathology corpus. The number of 1,000 reports from SMC is mainly colonoscopy pathology reports which were generated during health checkups at SMC from January 2019 to July 2019. SMC and AUSOM corpora are describing identical anatomic locations (i.e., colon and rectum), hence, we believe that both corpora are having homogeneous characteristics and able to apply identical annotation schema.

The graphs from Samsung Medical Center colorectal pathology corpus. (A) is a histogram showing the most frequently observed histology with their differentiation and anatomic location. (B) is a pie chart showing the location with their observed histologies.


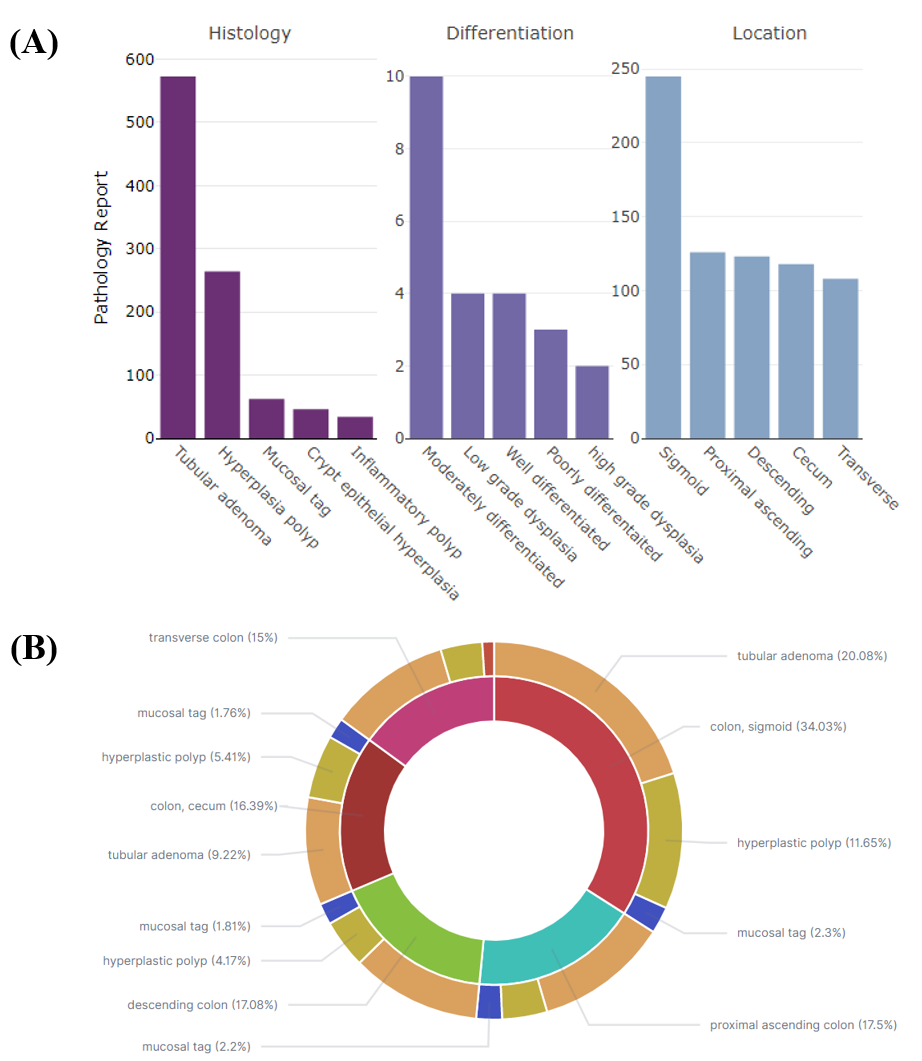

Supplement: Multimedia Appendix 5 [file medinform_v9i3e23983_app5.docx]
