# Supplementary material for: A Framework (SOCRATex) for Hierarchical Annotation of Unstructured Electronic Health Records and Integration Into a Standardized Medical Database: Development and Usability Study
Source: JMIR Med Inform. 2021 Mar 30;9(3):e23983. doi: 10.2196/23983 (PMC8044740; doi:10.2196/23983)
Supplement: Multimedia Appendix 6 [file medinform_v9i3e23983_app6.docx]

Multimedia Appendix 6. Annotation performance comparison between SOCRATex and traditional manual chart review

| **Annotation process** |  | **Sensitivity** | **Specificity** | **Precision** | **Accuracy** | **F1-score** | **Mean time (min.)** |
| --- | --- | --- | --- | --- | --- | --- | --- |
| Traditional Review |  |  |  |  |  |  |  |
|  | Annotator1 | 0.872 | 0.992 | 0.993 | 0.925 | 0.928 | 579 |
|  | Annotator2 | 0.848 | 0.995 | 0.996 | 0.909 | 0.916 | 516 |
|  | Average | **0.860** | **0.993** | **0.994** | **0.917** | **0.954** | **548** |
| SOCRATex |  |  |  |  |  |  |  |
|  | Annotator1 | 0.873 | 0.993 | 0.996 | 0.917 | 0.930 | 353 |
|  | Annotator2 | 0.919 | 0.976 | 0.989 | 0.937 | 0.953 | 366 |
|  | Average | **0.917** | **0.980** | **0.990** | **0.937** | **0.952** | **360** |
