# Supplementary material for: A Framework (SOCRATex) for Hierarchical Annotation of Unstructured Electronic Health Records and Integration Into a Standardized Medical Database: Development and Usability Study
Source: JMIR Med Inform. 2021 Mar 30;9(3):e23983. doi: 10.2196/23983 (PMC8044740; doi:10.2196/23983)
Supplement: Multimedia Appendix 7 [file medinform_v9i3e23983_app7.docx]

Multimedia Appendix 7. Comparison between SOCRATex and other NLP systems

| **Name** | **Full name (Description)** | **Input Data Type** | **Standardized Clinical Data Linkage** | **Supporting Schema Definition** | **Hierarchical Annotation** | **Word-level Information Extraction** | **Information Retrieval** | **User Interface** |
| --- | --- | --- | --- | --- | --- | --- | --- | --- |
| SOCRATex | Staged Optimization of Curation, Regularization, Annotation of clinical Text | OMOP-CDM/csv files | Y | Y | Y | N | Y | Y |
| EMERSE | Electronic Medical Record Search Engine | EHR | N | N | N | N | Y | Y |
| SemEHR | Surfacing Semantic Data from Clinical Notes in Electronic Health Records for Tailored Care, Trial Recruitment and Clinical Research | EHR | N | N | Y | Y | Y | Y |
| CREATE | Cohort Retrieval Enhanced by Analysis of Text from Electronic Health Records | OMOP-CDM | Y | N | N | N | Y | N |
| Sharma et al | Portable natural language processing based phenotyping system | OMOP-CDM | Y | N | N | N | N | N |
| cTAKES | Clinical Text Analysis Knowledge Extraction System | Text files | N | N | Y | Y | N | Y |
